# TNFSF13 Drives Atherosclerosis Progression by Targeting Macrophage Senescence

**DOI:** 10.7150/ijms.130617

**Published:** 2026-07-22

**Authors:** Xiao Lin, Mintong Liang, Shenglan Zeng, Kaiye Chen, Ying Yang, Fei Yu, Jiahui Zhou, Hui Tang, Hanqing Tang, Pengju Wen, Shulin Tang, Yueheng Wu

**Affiliations:** 1 The Second School of Clinical Medicine, Southern Medical University, Guangzhou, China.; 2 The First School of Clinical Medicine, Southern Medical University, Guangzhou, China.; 3 School of Basic Medical Sciences, Southern Medical University, Guangzhou, China.; 4 Medical Research Institute, Guangdong Provincial People's Hospital, Guangdong Academy of Medical Sciences, Southern Medical University, Guangzhou, China.; 5 Department of Cardiology, Guangdong Cardiovascular Institute, Guangdong Provincial People's Hospital, Guangdong Academy of Medical Sciences, Southern Medical University, Guangzhou, China.; 6 School of Mathematics, Foshan University, Foshan, China.; 7 Guangdong Cardiovascular Institute, Guangdong Provincial Key Laboratory of South China Structural Heart Disease, Guangdong Provincial People's Hospital, Guangdong Academy of Medical Sciences, Southern Medical University, Guangzhou, China.; 8 Department of Cardiovascular Surgery, Guangdong Provincial People's Hospital, Guangdong Academy of Medical Sciences, Southern Medical University, Guangzhou, China.

**Keywords:** atherosclerosis, cellular senescence, single-cell RNA sequencing, spatial transcriptomics, machine learning, macrophage

## Abstract

**Background:**

Atherosclerosis (AS), a complex age-related disease characterized by arterial lipid plaque formation, remains poorly understood at the molecular level. Senescent cells, particularly macrophages, drive plaque progression, but key senescence-inducing genes and their mechanisms are unclear.

**Method:**

Senescence-related DEGs (SR-DEGs) were identified by intersecting differentially expressed genes (DEGs) from GSE28829 with senescence-related genes (SRGs) in CellAge genes. Machine learning algorithms prioritized hub genes, whose expression and diagnostic performance were subsequently visualized with box plots and receiver operating characteristic (ROC) curves in the training dataset GSE28829, and validation sets GSE163154 and GSE100927. Immune infiltration analysis compared early and advanced plaques. Single-cell RNA sequencing (scRNA-seq) and spatial transcriptomics (ST) mapped hub gene expression across cell types and plaque regions. TNFSF13 was further validated in clinical atherosclerosis samples using Quantitative Real-time PCR (RT-qPCR), immunohistochemistry (IHC) and multiplex Immunohistochemistry (mIHC).

**Results:**

Advanced plaques exhibited 74 SR-DEGs compared to early plaques. Machine learning identified six senescence-related hub genes. ScRNA-seq revealed macrophage enrichment of all six hub genes, aligning with immune infiltration results. Bayesian deconvolution confirmed macrophage compositional shifts. Cell-cell communication analysis implicated TNFSF13-associated pathways. Spatial transcriptomics localized TNFSF13 upregulation specifically to the plaque core region, rich in SPP1+ macrophages and foam macrophages. Critically, TNFSF13 upregulation was robustly validated in human coronary artery tissues.

**Conclusion:**

This study identifies TNFSF13 as a master regulator of macrophage senescence driving atherosclerosis progression. By leveraging multi-omics and machine learning, we pinpoint the spatial and cellular context of TNFSF13 within plaques and validate its clinical relevance. TNFSF13 represents a promising therapeutic target to disrupt senescence-mediated plaque advancement in AS.

## Introduction

Atherosclerosis is a chronic inflammatory disease. Known risk factors contributing to AS-related mortality include dysregulated lipoprotein metabolism, inflammation, endothelial cell dysfunction, and mitochondrial DNA damage and so on 1 2 3 4. In recent decades, growing attention has been directed toward the association between AS, a disease predominantly affecting the elderly population 5, and organismal aging as well as cellular senescence, with the expectation of driving further research and therapeutic advancements in this field.

Aging is considered to be an independent risk factor for AS 6 7. Cellular senescence, a cellular state induced by endogenous or exogenous stimuli, can modulate gene expression in various disorders, including atherosclerosis 8. It not only brings about the initiation of atherosclerosis but also the rupture of unstable plaques 9 10, thus increasing the risk of acute coronary syndrome such as myocardial infarction and stroke. Unlike normal cells, senescent cells promote inflammation 11 and abnormal lipid metabolism 12. Interestingly, chronic inflammation and lipid metabolism disorders can conversely contribute to cellular senescence 13. Although some studies in recent years have revealed few aging markers in the development of atherosclerosis, there are still large gaps in the expression of senescence-related genes and their cellular distribution in AS 14 15 16.

Single-cell RNA sequencing technology is an advanced technology in the realm of cardiovascular disease, enabling high-resolution analysis of individual cell transcriptomes 17,18. Several studies have introduced scRNA-seq technology into the atherosclerosis field to explore the cellular functions of different cell types 19,20,21. However, substantial spatial information at the cellular level is lost due to the tissue dissociation during single-cell sequencing analysis. Therefore, to overcome the above limitation, we integrated spatial transcriptomics analysis with single-cell RNA sequencing technology. This integrated analysis can not only define a more comprehensive picture of cell-cell interactions within the atherosclerosis, but also characterize the tissue-level spatial patterns of the senescence-related genes.

To fill the gap, this study integrated bulk RNA sequencing method, scRNA-seq and spatial transcriptomics analysis to unravel the potential SR-DEGs in atherosclerosis, using the human coronary artery samples to further verify our conclusion. These data will shed light on the transcriptional landscape and phenotypic heterogeneity of senescent cells in atherosclerosis and open up new opportunities to explore different cell populations and their functions in atherosclerosis, thereby laying groundwork for developing new gene-targeting drugs.

## Materials and Methods

### Datasets

Gene expression datasets for atherosclerosis patients, including GSE28829, GSE163154, GSE100927, were obtained from the Gene Expression Omnibus (GEO, https://www.ncbi.nlm.nih.gov/geo/). The detailed information of the datasets is given in Supplementary Table 1.

### Obtaining differentially expressed genes and senescence-related genes

Based on the principal component analysis (PCA) results, four outlier samples were excluded from the GSE28829 dataset. A secondary PCA visualization was subsequently generated using the refined dataset, confirming the elimination of sample heterogeneity. All downstream analyses were consistently performed on this refined dataset to ensure analytical reliability. Differentially expressed genes within dataset GSE28829 were screened using “limma” package (version 3.58.1) with *p*-value < 0.05 and |Log2 fold-change (log2 FC) |≥ 0.5 in R project (R version 4.4.2). The DEGs were screened and visualized by heatmap and volcanic plot using “ggplot2” package to demonstrate the difference between two groups. The SRGs were obtained from CellAge database. Then the DEGs and the SRGs were intersected and the result was visualized using “ggvenn” package (version 0.1.10) to identify the SR-DEGs.

### Optimizing core hub genes by machine learning

Three advanced machine-learning algorithms—Least Absolute Shrinkage and Selection Operator regression (Lasso regression), Support Vector Machine-Recursive Feature Elimination (SVM-RFE) and Random Forest (RF) were deployed to screen out the optimal hub genes in the GSE28829 dataset. Six hub genes shared by the three models were identified for further in-depth analysis and visualized by a Venn plot. Hub genes identified in the GSE28829 dataset were individually modeled using generalized linear model (GLM) with subsequent ROC curve analysis. The predictive performance of these genetic models was systematically validated across two independent cohorts (GSE163154, GSE100927), demonstrating consistent discriminative power through comparative area under the curve (AUC) evaluations.

### Immune cell infiltration

The “CIBERSORT” package (version 0.1.0) was adopted to evaluate the relative infiltrate abundance of immune cells between the early atherosclerosis group and the advanced atherosclerosis group. The LM22 file was used as a reference expression signature of 22 types of immune cells. Only data with a p-value < 0.05 were reserved. Then, the “ggplot2” package was used to visualize the content of 22 types of immune cells in the atherosclerosis carotid artery. Additionally, box plots were used to show the difference of each immune cell between two groups. Finally, “ComplexHeatmap” package (version 2.22.0) was applied to visualize the correlation between immune cells and hub genes.

### Functional enrichment analysis of DEGs and SR-DEGs

To explore the functions and the pathways of DEGs and SR-DEGs, “clusterProfiler” package (version 4.10.0) was used to conduct GO and KEGG analysis. Gene ontology (GO) consists of genes' biological processes (BP), cellular components (CC), and molecular functions (MF). Kyoto Encyclopedia of Genes and Genomes (KEGG) is a database that integrates genomics, the biological process and disease information.

### Gene Set Enrichment Analysis (GSEA)

To further understand the potential role of DEGs, we performed the Gene set enrichment analysis (GSEA). The annotated gene set “h.all.v2025.1.Hs.symbols.gmt” was selected as reference gene set. Normalized Enrichment Score (NES) and False Discovery Rate (FDR) were calculated utilizing the R package clusterProfiler (version 4.14.4). Significantly enriched pathways were screened based on p-value < 0.05. The analyzed result was graphically presented using the R package ggplot2 (version 4.0.1).

### Single-cell transcriptome analysis

Single-cell sequencing data was downloaded from the CELLxGENE (https://cellxgene.cziscience.com/collections/db70986c-7d91-49fe-a399-a4730be394ac 22). Within the integrated and annotated publicly available single-cell datasets comprising 259,721 cells, cell cluster annotation outcomes were visualized through Uniform Manifold Approximation and Projection (UMAP) analysis, with subsequent employment of UMAP projections to delineate expression patterns of hub genes. The “CellChat” package (version 2.1.2) was used to conduct the intercellular communications revealing the relationship between different cells and their interplay in specific pathway. “BayesPrism” package (version 2.2.2) was employed to deconvolve bulk RNA-seq profiles into cellular proportions which were identified at the single-cell level.

### Spatial transcriptomics profiling

Spatial transcriptomics data was downloaded from Zenodo (https://zenodo.org/ records/14007461^23^).

Based on the integrated and annotated publicly available spatial transcriptomic dataset, histopathological features in three representative tissue sections were visualized spatially and the spatial expression patterns of hub genes in these pathological specimens were further characterized.

### Tissue collection

A total of four AS left coronary arteries were harvested from patients with severe atherosclerotic left coronary artery diseases who received heart transplantations at Guangdong Provincial People's Hospital. Four pairs of normal left coronary arteries were obtained from organ donors at the time of organ procurement. The collected samples were respectively frozen in liquid nitrogen and fixed with paraformaldehyde.

### Quantitative real-time PCR

Total RNA was extracted from four atherosclerosis-affected coronary arteries and four normal coronary arteries, using the RNeasy® Mini Kit (50) (Thermo Fisher Scientific, Waltham, MA, USA, No. 74904) and reverse-transcribed into complementary DNA (cDNA) using a PC-96 Gradient PCR Instrument (Yooninf, Hangzhou, China; PC-96). After that, RT-qPCR was performed using the CFX96 Touch Real-Time PCR Detection System (Bio-Rad, Hercules, CA, USA). PCR conditions were as follows: 95℃ for 30 seconds, followed by 40 cycles at 95℃ for 3 seconds and 60℃ for 30 seconds. RT-qPCR was performed in triplicate, and the average expression of mRNA was calculated using the 2^-ΔΔCt^ method. The primer sequences for TNFSF13 and GAPDH are listed in Supplementary Table 2.

### Immunohistochemistry (IHC)

Dewax and rehydrate the paraffin-embedded aortic tissue sections, then add 10 mmol/L citrate buffer solution for antigen retrieval. Subsequently, 3% H_2_O_2_ was added and incubated for 10 minutes to reduce the influence of endogenous peroxidase activity. Then 5% BSA was added and incubated at 37 °C for 30 minutes to block non-specific binding sites. After that, incubate the sections with primary antibodies TNFSF13 (ab189263, abcam, Shanghai, China) at 4 °C overnight. The sections were then washed with PBS three times, 5 minutes each. Subsequently, the sections were incubated with secondary antibody goat anti-rabbit IgG-HRP (Solarbio, Beijing, China) at 37 °C for 90 minutes followed by three washes with PBS, 5 minutes each. Then the sections are stained with DAB chromogenic reagent and counterstained with hematoxylin. The sections are observed under a microscope and processed with ImageJ software.

### Multiplex Immunohistochemistry (mIHC)

Upon collection, left coronary artery specimens were immediately fixed in formalin for 24-48 hours. Subsequent processing included standard procedures of tissue dehydration, paraffin embedding, and sectioning. Paraffin sections were baked at 65 °C for 4 hours, followed by deparaffinization and rehydration through xylene and graded alcohol series. Antigen retrieval was performed using EDTA buffer (pH 9.0). Sections were then blocked with an antibody diluent to reduce nonspecific binding. Immunostaining was carried out by sequential incubation with primary and secondary antibodies according to the experimental protocol. Target antigens included TNFSF13 (ab189263, Abcam, Shanghai, China), CD68 (ab316218, Abcam, Shanghai, China), iNOS (ab283655, Abcam, Shanghai, China) and CD163 (ab156769, Abcam, Shanghai, China), and cell nuclei were counterstained with 4′,6-diamidino-2-phenylindole (DAPI).

### Ethics declarations

#### Ethics approval

The study was approved by the Ethics Committee of the Guangdong Provincial People's Hospital (GDREC2016255H) and all patients gave informed consent for the use of their tissue samples. The research is performed in accordance with the Declaration of Helsinki principles.

#### Consent to participate

All participants or their legal guardian received comprehensive study information, including its objectives, procedures, potential risks, and benefits. Written informed consent was obtained from all participants or their legal representatives following this detailed explanation. Personally identifiable information was anonymized, and the study results did not contain any information that could be traced back to an individual.

## Results

### The genes of the early-stage atherosclerotic plaques are different from those of the advanced plaques

**Figure 1:**
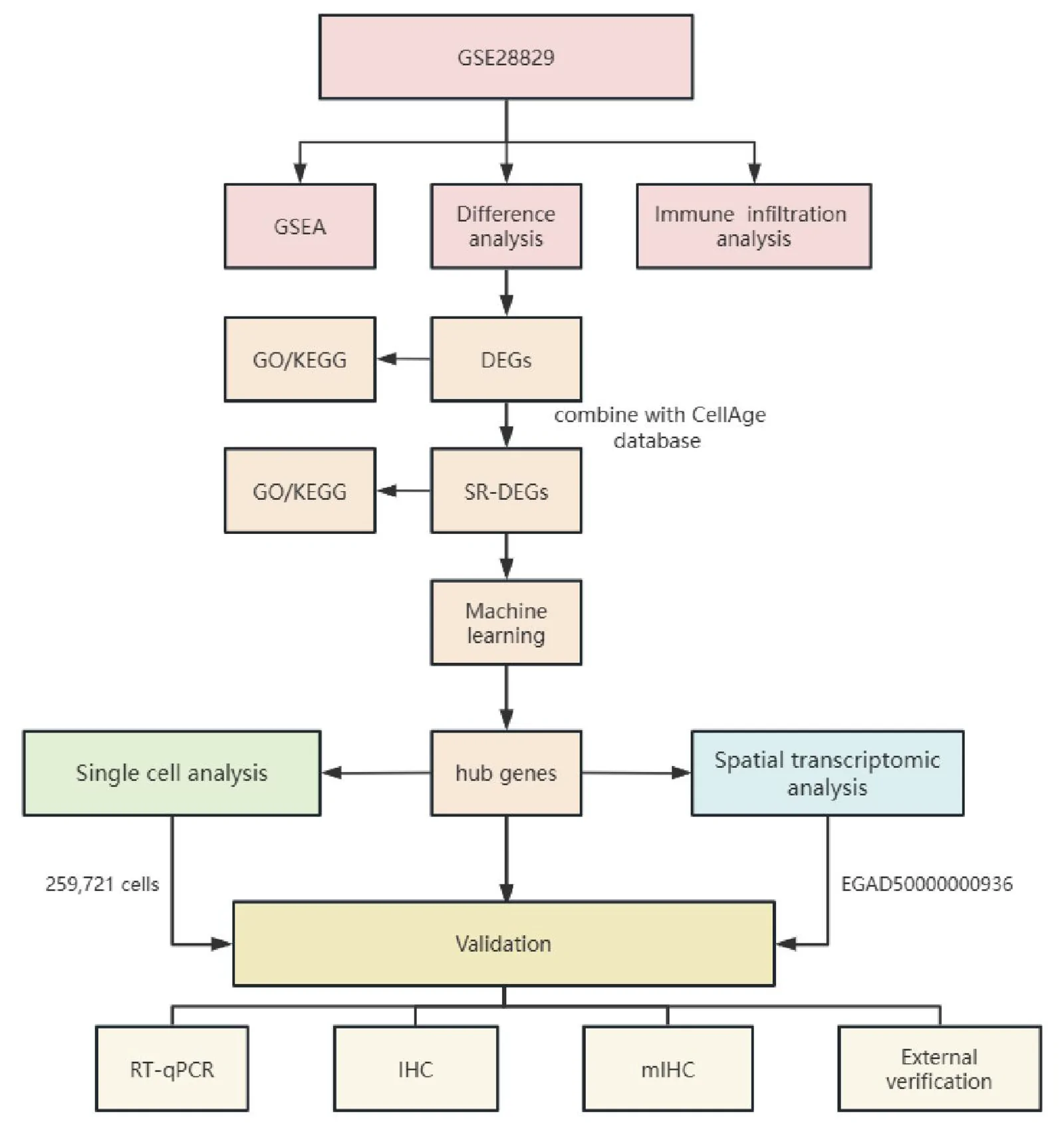
Illustration of the data analysis workflow and experiment procedure.

**Figure 2:**
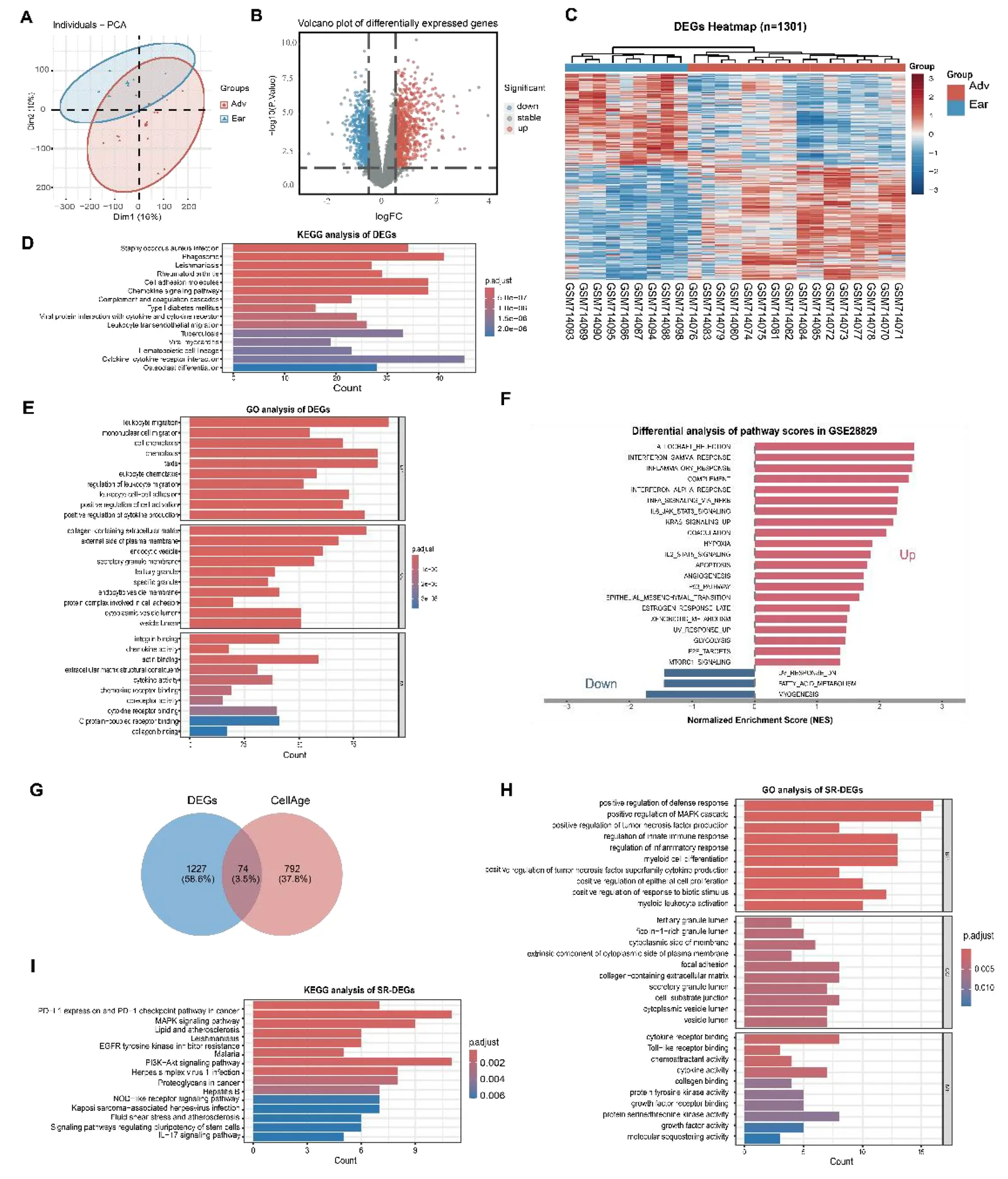
Transcriptomic characteristics of advanced atherosclerotic lesions. (A). Transcriptomic landscape segregation through principal component analysis; (B). Volcano plot of DEGs between early atherosclerotic plaque and advanced atherosclerotic plaque group; (C). Hierarchically clustered heatmap of 1301 differentially expressed genes. The markedly upregulated DEGs were labeled in red and the markedly downregulated DEGs were labeled in blue(|log2FC|>0.5, *p*<0.05); (D). Pathway activation landscape displaying the top 15 enriched KEGG pathways of DEGs in dataset GSE28829; (E). Functional annotation hierarchy showing top enriched terms: top 10 biological processes (BP), cellular components (CC), and molecular functions (MF) of DEGs in dataset GSE28829 by GO analysis; (F). GSEA enrichment pathways in dataset GSE28829 (G). Intersection of DEGs (1301 genes) and SRGs (866 genes) using Venn visualization; (H). Functional annotation hierarchy showing top enriched terms: top 10 biological processes (BP), cellular components (CC), and molecular functions (MF) of SR-DEGs in GO analysis; (I). Pathway activation landscape displaying the top 15 enriched KEGG pathways of SR-DEGs.

The overview of the study is depicted in Figure 1. To systematically characterize the transcriptional alterations during atherosclerotic plaque progression, we performed RNA sequencing analysis on the GSE28829 cohort. Principal component analysis (PCA) revealed clear segregation between advanced atherosclerotic plaque (Adv) and early atherosclerotic plaque (Ear) (Fig. 2A), following appropriate data normalization (Fig. S1). Our analysis identified 1,301 statistically significant differentially expressed genes (*p-*value < 0.05, |log2FC|> 0.5), of which 729 genes were markedly upregulated and 572 genes were markedly downregulated (Fig. 2B). To further elucidate these transcriptional differences, we also employed a heatmap to demonstrate the hierarchical clustering of all significant DEGs (Fig. 2C). KEGG enrichment analysis of DEGs revealed significant enrichment of Chemokine signaling pathway and Cytokine-cytokine receptor interaction, which is related to atherosclerosis progression (Fig. 2D). Similarly, GO analysis of DEGs also showed that pathways associated with plaque stability and immune regulation such as leukocyte migration, mononuclear cell migration, chemokine activity, cytokine activity, collagen-containing extracellular matrix and extracellular matrix structural constituent were significantly upregulated (Fig. 2E). Meanwhile, we also performed Gene Set Enrichment Analysis (GSEA) showing significant upregulation of pathways such as inflammatory response, IL-6-JAK-STAT3 signaling and p53-pathway (Fig. 2F). These comparative analyses reveal significant differences in gene expression patterns between the early and advanced plaques and indicate the driving role of aging during AS plaque progression.

### The SR-DEGs have diverse biological functions and participate in multiple biological pathways

Guided by the above objective, we intersected DEGs and SRGs to further investigate the relationship between aging and atherosclerosis. We identified 74 SR-DEGs shown in the Venn plot in Fig. 2G. After that, the potential mechanistic roles of SR-DEGs in AS pathogenesis were elucidated through systematic functional annotation including GO and KEGG pathway analyses. Biological process enrichment was predominantly associated with positive regulation of defense response, positive regulation of MAPK cascade and positive regulation of tumor necrosis factor production. Cellular component analysis identified tertiary granule lumen, ficolin-1-rich granule lumen and cytoplasmic side of membrane as predominant sites of action. Molecular functions were concentrated in cytokine receptor binding, Toll-like receptor binding, and chemoattractant activity (Fig. 2H). Pathway enrichment analysis through KEGG database interrogation identified significant involvement of PD-L1 expression and PD-1 checkpoint pathway in cancer, MAPK signaling pathway, and Lipid and atherosclerosis (Fig. 2I).

### Hub genes were identified through machine learning

**Figure 3:**
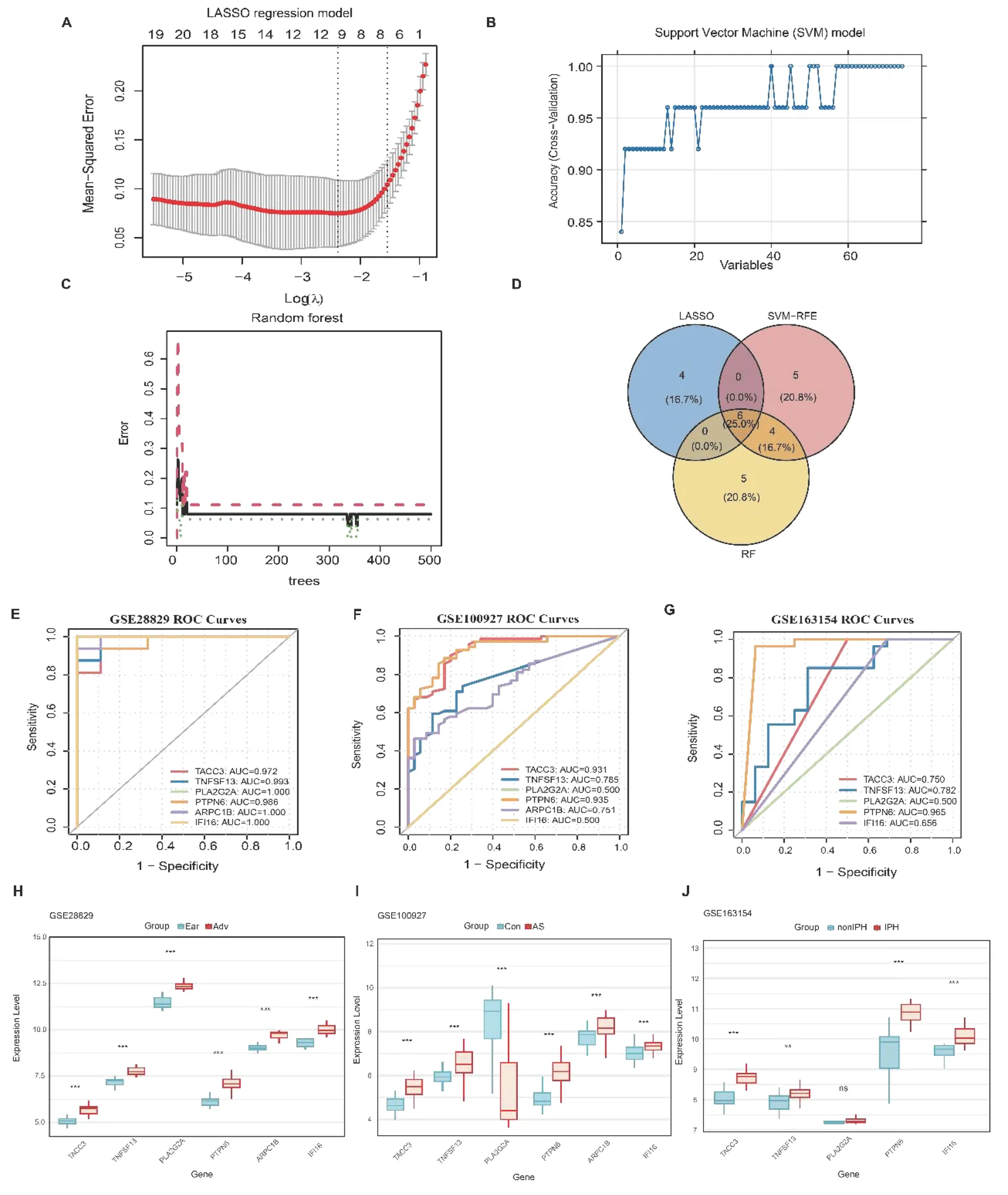
Identification of hub genes through machine learning. (A). Lasso regression analysis for Optimal Lambda Selection. (B). SVM-RFE error rate curve indicating the optimal feature subset. (C). Random Forest variable importance plot for top-ranked genes. (D). Venn diagram illustrates six shared genes identified by LASSO, SVM-RFE and Random Forest. (E)-(G). ROC curve and 95% CI of six hub genes in GSE28829, GSE100927 and GSE163154, demonstrating their predictive performance quantified by AUC values. (H)-(J). Boxplots of expression differences of six hub genes between AS and normal groups in GSE28829, GSE100927 and GSE163154 cohorts, respectively. The significance was all defined as *p<0.05, **p<0.01, ***p<0.001.

After detecting 74 SR-DEGs at the intersection of DEGs and known aging markers (Fig. 2A), we applied LASSO, SVM-RFE, and Random Forest to select hub genes. Lasso regression algorithm with 5-fold cross-validation selected 10 genes based on the minimum Mean-Squared Error criterion (Fig. 3A). The SVM-RFE algorithm, implemented with 5-fold cross-validation, systematically identified the top 15 genes (Fig. 3B). The Random Forest algorithm was employed to select the top 15 genes based on their importance scores (Fig. 3C). Finally, we combined the above algorithms and identified 6 hub genes in AS, including TACC3, TNFSF13, PLA2G2A, PTPN6, ARPC1B and IFI16 as presented in the Venn plot (Fig. 3D). Subsequently, we examined the effectiveness of six hub genes in predicting AS by constructing ROC curves and calculating the AUC and 95% confidence intervals (CI) in the training cohort (GSE28829) and validate their predicting efficacy in two other independent cohorts (GSE100927 and GSE163154) (Figs. 3E-G). After this verification, we performed differential expression validation of these six hub genes across all three datasets. Five genes (TACC3, TNFSF13, PTPN6, ARPC1B and IFI16) exhibited significant upregulation in all datasets, while PLA2G2A showed significant downregulation specifically in the GSE100927 dataset (Figs. 3H-J). Overall, TNFSF13, TACC3 and PTPN6 demonstrates remarkable diagnostic value for AS. The resulting heatmap reveals strong positive correlations among the six hub genes. Correlation heatmap of six hub genes indicates that they exhibit similar expression patterns across samples (Fig. S2).

### Macrophages M0 and macrophages M2 are the dominate immune cells in advanced atherosclerotic plaque and highly related to hub genes

**Figure 4:**
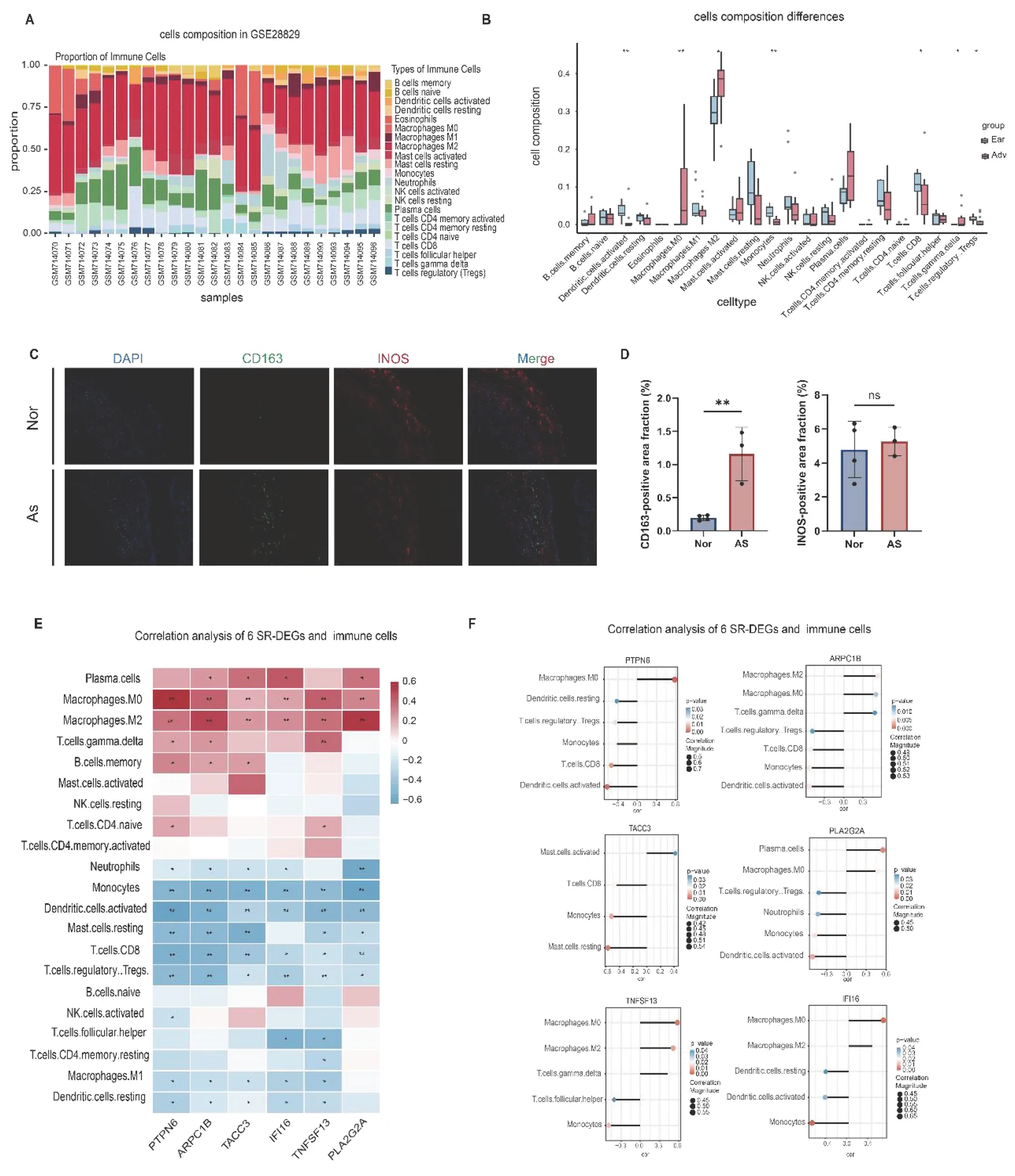
Immune cell infiltration and correlation analysis. (A). Heatmap of composition of 22 types of immune cell in GSE28829. (B). Boxplot of 22 immune cell composition between early atherosclerotic plaque (Ear) and advanced atherosclerotic plaque (Adv) in GSE28829. Blue indicates Ear group and red indicates the Adv group. (C). Representative mIHC images show the expression of CD163 and INOS in the normal and AS group. Scale bar=50 µm. (D). The bar plots showing the positive area fraction of CD163 and INOS in mIHC. (E). Correlation heatmap of 6 hub genes and the abundance of immune cells in AS. Blue indicates negative correlation, red indicates positive correlation. (F). The dot plot shows the correlation analysis of 6 hub genes and the abundance of immune cells in AS. The red indicates more significant while blue indicates less significant. The size of the dot indicates the correlation magnitude. The significance was all defined as *p<0.05, **p<0.01, ***p<0.001.

Since AS is highly associated with immunology, we conducted immune cell infiltration analysis to investigate the relationship between SR-DEGs and immune cells. We performed CIBERSORT algorithm to analyze the 22 immune cell phenotypes in each sample of GSE28829 (Fig. 4A). Compared with early atherosclerotic plaques, advanced atherosclerotic plaques exhibited a higher proportion of macrophages M0, macrophages M2 and T cells gamma delta and a lower proportion of Dendritic cells activated, Monocyte, T cells CD8 and T cells regulatory (Tregs) (Fig. 4B). As shown by mIHC analysis, M2 macrophage marker CD163 signals were sparse in normal coronary arteries but significantly more abundant in atherosclerotic tissues (Fig. 4C). The box plot indicated a substantial rise in the positive area fraction of CD163 within the atherosclerotic group (Fig. 4D). However, the expression levels of M1 macrophage marker iNOS remained comparable between the two groups, showing no statistically significant variation (Fig. 4C-D). Furthermore, we delved deeper into exploring the associations between 6 hub genes and the abundance of immune cells using Spearman's correlation analysis and showed only immune cells with statistically significant genetic associations in Figure. 4E. According to the results, TNFSF13 was positively related to macrophages M0 (cor = 0.580, *p* < 0.01) as well as macrophages M2 (cor = 0.515, *p* < 0.01) and negatively related to Monocyte (cor = -0.493, *p* = 0.012). PTPN6 was positively related to macrophages M0 (cor = 0.781, *p* < 0.01) and negatively related to Dendritic cells activated (cor = 0.630, *p* < 0.01). TACC3 was negatively related to Mast cells resting (cor = -0.569, *p* < 0.01). PLA2G2A was positively related to plasma cells (cor = 0.549, *p* < 0.01) and negatively related to Dendritic cells activated (cor = -0.509,* p* < 0.01). IFI16 was positively related to macrophages M0 (cor = 0.615, *p* < 0.01) and negatively related to Monocyte (cor = -0.654, *p* < 0.01). Interestingly, 6 hub genes all have a strong positive correlation with macrophages M0 and macrophages M2 (*p* < 0.01) and negative correlation with Dendritic cells activated and monocyte (*p* < 0.01) (Fig. 4F). Overall, there are obvious differences of immune cell composition between early and advanced plaque groups, and hub genes are highly related to monocytes and macrophages in AS.

### Single-cell RNA-seq analysis of cellular expression of 6 hub genes in AS

**Figure 5:**
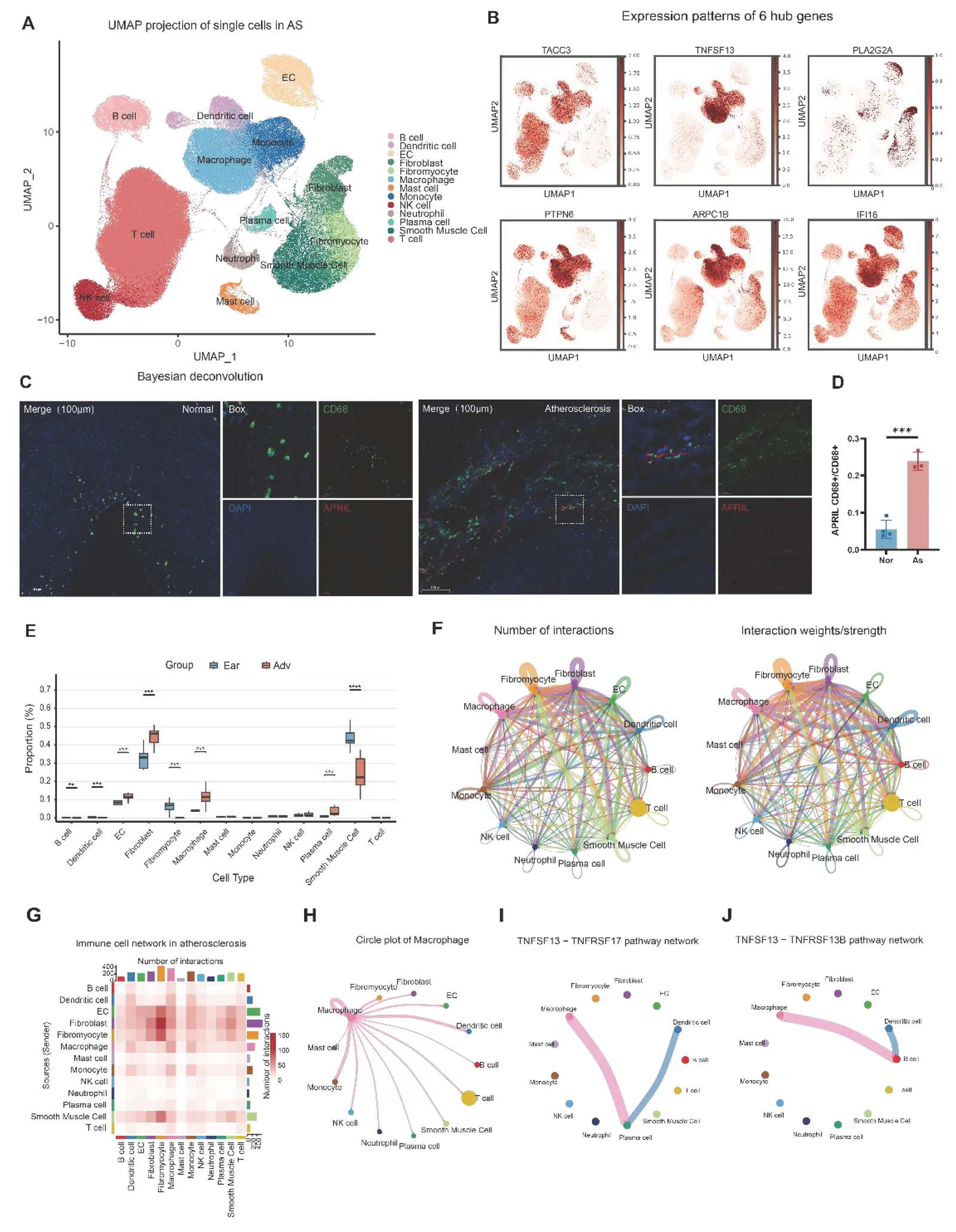
Single-cell RNA-seq analysis and cell-cell communication in AS. (A). The cluster of 13 types of cells include B cells, fibromyocytes, NK cells, dendritic cells, macrophages, neutrophil, endothelial cells, mast cells, plasma cells, fibroblasts, monocytes, smooth muscle cells and T cells. (B). Localization and expression patterns of six hub genes in 13 cell clusters. (C). Representative images of antibody staining of TNFSF13 between normal and atherosclerosis groups in mIHC. Scale bars, 100μm. (D). The bar plot showing the proportion of macrophages expressing TNFSF13 in mIHC. (E). Bayesian deconvolution analysis showing alterations in cellular composition between the Ear and Adv groups in GSE28829. (F). The interaction net counts plot of different cells in AS. The thicker line represented more interactions and stronger interaction weights between two cells. (G). Heatmap showing the interaction between 13 types of cells based on ligands-receptors principle. On the top of heatmap, the higher the pillar is, the greater the total number of interactions that the cell has with other cells as a sender; On the right side, the higher the pillar is, the greater the total number of interactions that the cell has with other cells as a receiver. (H). The circle plot showing the interaction of macrophages and other cells. (I)-(J). Intercellular communication networks. The circle plot showing the intercellular communication network for TNFSF13-TNFRSF17(I) and TNFSF13-TNFRSF13B(J). The significance was all defined as *p<0.05, **p<0.01, ***p<0.001.

To elucidate the cellular and molecular mechanisms underlying six hub genes at single-cell resolution, we performed an integrative analysis of their cellular localization and expression patterns of six hub genes across 259,721 single-cell transcriptomes encompassing 13 distinct cell populations: B cells, macrophages, plasma cells, dendritic cells, mast cells, fibroblasts, endothelial cells, monocytes, T cells, NK cells, neutrophil, smooth muscle cells, and fibromyocytes (Fig. 5A). TACC3 was predominantly expressed in macrophages, monocytes, dendritic cells, B cells, T cells, and NK cells. PLA2G2A exhibited elevated expression in fibroblasts and endothelial cells, while PTPN6 demonstrated broad expression across macrophages, monocytes, dendritic cells, B cells, T cells, NK cells, plasma cells, and mast cells. ARPC1B was highly expressed in macrophages, monocytes, dendritic cells, and endothelial cells. IFI16 displayed the most extensive expression pattern, spanning macrophages, monocytes, dendritic cells, B cells, T cells, NK cells, endothelial cells, and fibroblasts. Notably, TNFSF13 was specifically highly expressed in macrophages (Fig. 5B). Consistent with this, mIHC analysis revealed a significantly higher proportion of TNFSF13-positive macrophages in atherosclerotic plaques compared to the control group (Figs. 5C-D), suggesting that TNFSF13 may influence atherosclerosis via macrophages.

To further compare the cellular landscape differences between the early atherosclerotic plaque and advanced atherosclerotic plaque groups, we performed Bayesian deconvolution of GSE28829 using the signature matrix derived from the aforementioned single-cell RNA-sequencing data. This analysis estimated the relative proportions of various cell populations within the bulk dataset. The deconvolution analysis revealed significant alterations in cellular composition between the Ear and Adv groups (Fig. 5E). Specifically, the Adv group exhibited a significantly higher proportion of fibroblasts (p < 0.001), macrophages (p < 0.001), endothelial cells (EC) (p < 0.001), and plasma cells (p < 0.001). Conversely, the Ear group demonstrated elevated proportions of smooth muscle cells (p < 0.001), and fibromyocytes (p < 0.001). These findings suggest a profound remodeling of the cellular microenvironment in the atherosclerotic condition, characterized by an enrichment of cellular composition including macrophages and fibroblasts.

In order to investigate the potential target of TNFSF13 pathway, we conducted cell-cell communication analysis. There are extensive connections among immune cells in atherosclerosis (Fig. 5F). Among them, the secretory activity of macrophages, fibroblasts, and endothelial cells are the strongest, while fibroblasts, macrophages, endothelial cells, and smooth muscle cells receive more signals than other cells (Fig. 5G). This suggests that fibroblasts, macrophages, and endothelial cells are in a key position in atherosclerosis. Among them, the macrophages, in which the six hub genes are highly expressed, exhibited a strong association with other cells (Fig. 5H). More importantly, the plot of TNFSF13 pathway network showed that macrophage and dendritic cells secreted TNFSF13 and mainly acted on B cells and plasma cells, of which the receptors are also known as BCMA(TNFRSF17) and TACI(TNFRSF13B) (Figs. 5I-J).

### Spatial profiling of hub genes reveals distinct localization patterns and co-localization with pathological cell populations in atherosclerotic plaques

**Figure 6:**
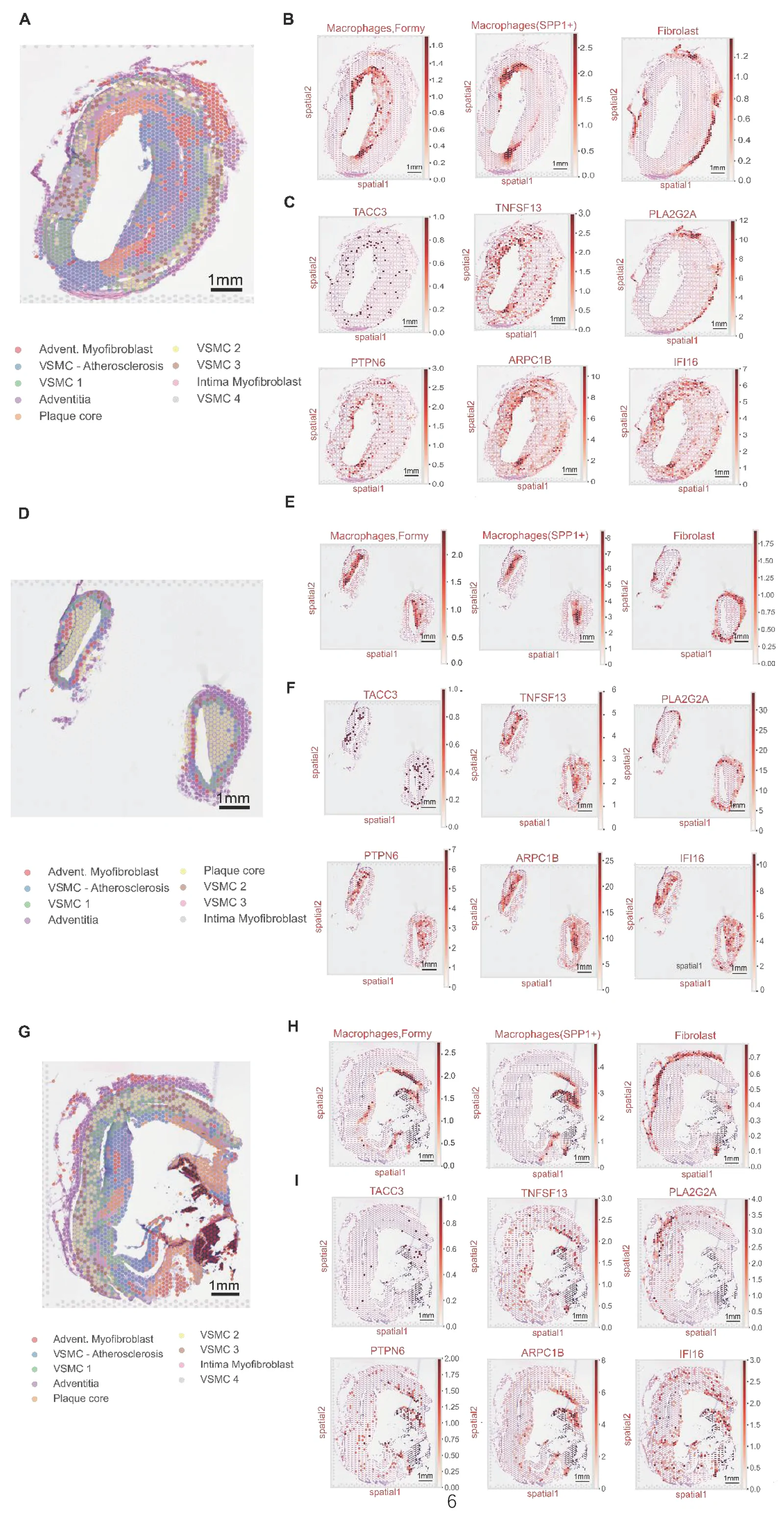
Spatially transcriptomic analysis of hub genes in AS. (A). (D). (G). Transcriptional annotation of atherosclerosis coronary artery slides. (B). (E). (H). Spatiotemporal mapping the macrophages, foamy cells and fibroblasts spatial distribution patterns in representative slides obtained from of atherosclerotic samples. (C). (F). (I). Spatiotemporal mapping the hub genes spatial distribution patterns in representative slides obtained from of atherosclerotic samples.

Building upon the identification of hub genes across distinct cellular clusters, we aimed to investigate their spatial expression heterogeneity and map the co-localization of hub genes with these pathological cell populations within pathologically relevant tissue zones. To achieve this, we leveraged spatial transcriptomics using a publicly available dataset from Bleckwehl et al, whose spatial spots had been computationally clustered and annotated into nine distinct regions, such as Adventitia and Plaque core (Figs. 6A, D and G). We systematically characterized the localization of six hub genes in relation to specific pathologically relevant tissue zones. Analysis of three atherosclerosis slides demonstrated that the macrophages, foamy macrophages and SPP1^+^ macrophages were mainly located in the plaque core region and the fibroblasts were in the adventitia areas (Figs. 6 B, E and H). It also revealed that TNFSF13, TACC3, PTPN6, IFI16 and ARPC1B exhibited elevated expression within plaque core regions of atherosclerotic lesions, whereas PLA2G2A demonstrated predominant localization in adventitia areas. (Figs. 6C, F and I).

### TNFSF13 was upregulated in atherosclerosis tissues

**Figure 7:**
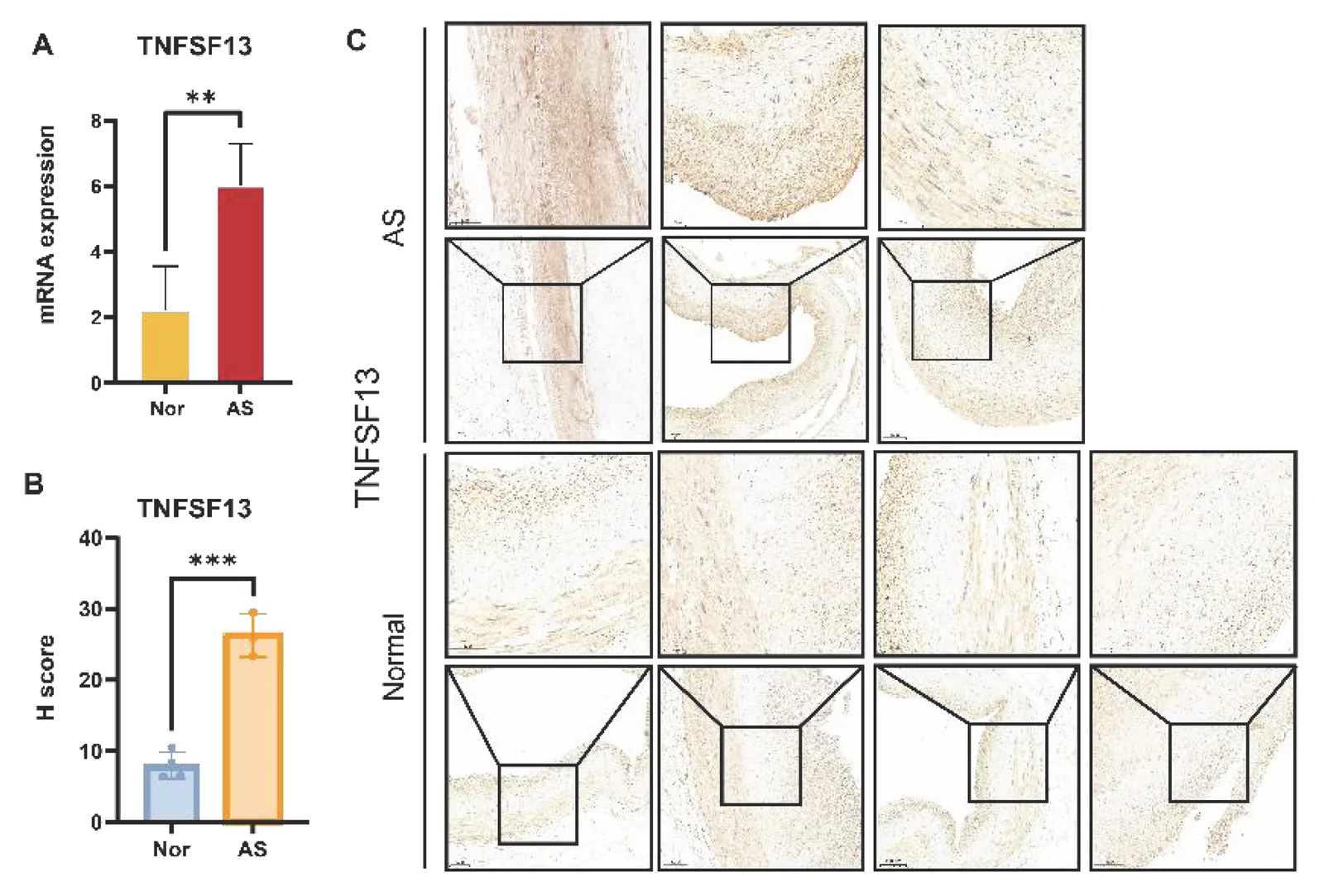
RT-qPCR and IHC show upregulated expression of TNFSF13 in atherosclerosis tissues. (A). The bar chart of TNFSF13 mRNA expression level in coronary artery comparing normal and AS patients. (B). The bar chart of TNFSF13 level in aorta slides comparing normal and AS patients. (C). Representative images of TNFSF13 expression in aortic roots of human with normal and AS aorta stained by H&E and DAB chromogenic reagent. Scale bar:100 μm and 50μm.The significance was all defined as *p<0.05, **p<0.01, ***p<0.001.

To further explore the expression profiles and potential functional implications of TNFSF13 (APRIL) in atherosclerotic and normal tissues, we performed RT-qPCR to quantify its mRNA expression levels between the atherosclerotic and normal groups. The RT-qPCR results revealed that the mRNA expression level of TNFSF13 in the atherosclerotic group was significantly higher than the normal group (P < 0.01) (Fig. 7A). IHC was further extended to provide spatial localization and protein-level evidence supporting the differential expression patterns of TNFSF13 in atherosclerosis compared with normal tissues. The Histochemistry score (H score) of IHC revealed that the expression level of TNFSF13 in the atherosclerotic group was significantly higher than that in the normal group (p < 0.05) and was mainly enriched in the inner region of the plaque (Figs. 7B-C). These observations, consistent with the transcriptomic and spatial transcriptomic analyses, indicated that human atherosclerotic plaque had a remarkable increase in TNFSF13 both in protein and mRNA levels and revealed its potential role in promoting the progression of atherosclerosis.

## Discussion

Atherosclerosis is a chronic progressive disease characterized by the formation of subintima atherosclerotic plaques. Early stable atherosclerotic plaques usually do not cause serious damage to the body. However, under the influence of various factors, the stable plaques will transform into unstable plaques, inducing an acute cardiovascular response. Cellular senescence has been established as an independent contributing factor to atherosclerosis, accelerating both disease progression and plaque rupture. Senescent cells release senescence-associated secretory phenotype (SASP), including cytokines and matrix metalloproteinases (MMPs). Previous studies have demonstrated the presence of senescent cells throughout all stages of atherosclerotic plaque formation, including endothelial cells, macrophages, and smooth muscle cells. Mechanistically, it has also been reported that dyslipidemia-induced cellular senescence promotes AS pathogenesis 13, while mitigation of oxidative stress can attenuate cellular senescence and slow AS progression 24. Previous studies exploring senescence-associated genes in the progression of AS have mostly focused on individual cell 25 26, which may have overlooked the overall aging characteristics of the plaque, including the spatial heterogeneity of cellular distribution within AS plaques, the contribution of senescence-associated genes across different cell types, and the intercellular interactions among senescent cells. Here, we found that senescence-associated gene TNFSF13 have high diagnostic value and may promote AS plaque formation by promoting macrophage senescence. These data indicate that cellular senescence may play a vital role in atherosclerosis and may represent a potential therapeutic target. Our findings provide a more comprehensive identification of SR-DEGs in AS.

Through the above bioinformatics analysis and machine learning methods, six key candidate genes were identified: TACC3, TNFSF13, PLA2G2A, PTPN6, ARPC1B, and IFI16. TACC3 is a multifunctional protein of the transforming acidic coiled-coil protein (TACC) family, which is associated with cell proliferation and tumor metastasis through the activation of the PI3K/Akt and ERK signaling pathways 27. PLA2G2A, a key member of the Secretory Phospholipase A2 (sPLA2) family, exerts a critical function in the early stage of atherosclerosis 28. PTPN6 (Protein Tyrosine Phosphatase Non-Receptor Type 6) can promote VitD3-induced macrophage autophagy and exhibit dual regulatory roles in inflammatory diseases such as atherosclerosis 29. ARPC1B is one of the subunits of the Arp2/3 complex associated with the cytoskeleton, and its deficiency is linked to multisystem diseases such as platelet abnormalities 30. Interferon-γ-inducible protein 16 (IFI16) plays a critical role in immune responses and in multiple cellular death mechanisms 31.

TNFSF13 (A Proliferation Inducing Ligand, APRIL), a member of the tumor necrosis factor superfamily, plays a critical role in regulating B cell function and promoting tumor cell growth 32. TNFSF13 can be secreted by a variety of cells such as platelets 33, neutrophils, and macrophages and interact with the receptors such as transmembrane activator and CAML interactor (TACI), B-cell maturation antigen (BCMA) and heparan sulfate proteoglycan (HSPG). In the realm of cardiovascular field, it is considered to be a biomarker associated with atrial fibrillation 34 and heart failure 35. In recent years, the anti-atherosclerotic effect of APRIL through binding to HSPG has also gained widespread attention 36. These findings revealing that APRIL may also have pathophysiological and physiological significance in atherosclerosis.

During cellular senescence, a broad array of SASP components is produced, including pro-inflammatory cytokines and MMPs. The expression of these SASP components is primarily regulated by the MAPK and NF-κB signaling pathways 37 and contributes to chronic inflammation and immune dysfunction in atherosclerosis 38. Senescent macrophages, as key cells producing SASP, have been found to accumulate in atherosclerosis. Furthermore, previous studies have revealed that activation of the TNFSF13 receptor can induce inflammatory activation of macrophages via the MAPK and NF-κB pathways 39. Therefore, we speculate that TNFSF13 may regulate the secretion of SASP by macrophages in atherosclerosis through the MAPK and NF-κB pathways, thereby influencing the progression of chronic inflammation in AS. In our study, GO and KEGG analyses indicated significant enrichment of the MAPK pathway. Concurrently, we found that TNFSF13 was significantly upregulated in atherosclerosis, which is consistent with previous results, and it was predominantly enriched in macrophages. This suggests that TNFSF13 may play a critical role in atherosclerosis by modulating chronic inflammatory responses in macrophages.

Immune cells play multiple roles during the progression of atherosclerosis. In our study, the immune infiltration results showed the highest percentage of macrophages and T cells within the plaques, which is in line with previous studies 40. In addition, we found that progressive plaques had a significantly higher proportion of M0 macrophage, M2 macrophage, and γδ T cells, which is consistent with previous findings by Jia Gao et al 41. However, no significant differences were found in macrophage M1 composition between the two groups. In the progression of atherosclerosis, M1 macrophages may promote the inflammatory response around the plaque to accelerate plaque rupture, while M2 macrophages tend to maintain plaque stability in the early stage of plaque formation 40. γδ T cells influence atherosclerosis through the production of IL-17, but they exhibit no significant effect on the early progression of AS 42 43. Moreover, correlation analysis demonstrated that TNFSF13 was highly correlated with macrophage M2. These findings suggest that TNFSF13 is highly related to immune cells and that it may influence the progression of atherosclerosis by modulating these cellular functions.

Subsequently, we assessed the distribution and expression of TNFSF13 at the single-cell and cell cluster levels using a single-cell high-throughput approach. Our results showed that TNFSF13 was mainly enriched in macrophages, monocytes, and dendritic cells. It is widely acknowledged that macrophages, especially foam cells, are the key to plaque formation. In the early stages of atherosclerosis, macrophages phagocytose and store lipoproteins to lower blood lipids. However, when macrophages phagocytose too much lipid or when lipid metabolism is disturbed, excessive lipid accumulation in the intracellular compartments will result in the formation of foam cells, which will accumulate to form plaques. Increasing autophagy, promoting macrophage exocytosis of lipids, and enhancing phagocytosis of apoptotic cells can delay plaque formation and expansion 44; whereas senescent macrophages impede plaque regression and increase plaque instability due to their reduced autophagic flux, mitochondrial dysfunction, and cellular dysfunction 45. These results suggest that the hub gene TNFSF13 may influence the progression of atherosclerosis by regulating macrophage function.

**In conclusion, we identified TNFSF13 as a senescence-associated biomarker in atherosclerosis and established a senescence-related diagnostic model. Through integrated multi-omics approaches, we systematically mapped the spatial distribution patterns of six senescence-associated genes and further deciphered their tissue-specific expression profiles and intercellular communication networks. However, there are several limitations of our study.** Although integrating scRNA-seq and spatial transcriptomic analysis identifies the key candidate genes and provides a theoretical landscape of their roles in the progression of atherosclerosis, the mechanisms by which TNFSF13 regulates macrophage senescence remain to be determined. Further experiments using conditional deletion or overexpression of TNFSF13 in relevant cell lines (such as VSMCs or macrophages) will help delineate the specific contribution of TNFSF13 to atherosclerosis.

## Supplementary Material

Supplementary figures and tables.

## Data Availability

Gene expression datasets for atherosclerosis patients, including GSE28829, GSE163154, GSE100927, were available in the Gene Expression Omnibus (GEO, https://www.ncbi.nlm.nih.gov/geo/). Single-cell sequencing data is available in the CELLxGENE (https://cellxgene.cziscience.com/collections/db70986c-7d91-49fe-a399-a4730be394ac1). Spatial transcriptomics data is available in Zenodo (https://zenodo.org/ records/14007461).

## Abbreviations

AS: atherosclerosis
DEGs: differentially expressed genes
SRGs: senescence-related genes
SR-DEGs: senescence-related differentially expressed genes
PCA: principal component analysis
IPH: intraplaque hemorrhage
non-IPH: non-intraplaque hemorrhage
SASP: senescence-associated secretory phenotype
GO: gene ontology
BP: biological processes
CC: cellular components
MF: molecular functions
KEGG: Kyoto encyclopedia of genes and genomes
Lasso regression: least absolute shrinkage and selection operator regression
SVM-RFE: support vector machine-recursive feature elimination
RF: random forest
GLM: generalized linear model
ROC: receiver operating characteristic
AUC: area under the curve
UMAP: uniform manifold approximation and projection
ScRNA-seq: single-cell RNA sequencing
ST: spatial transcriptomic
IHC: immunohistochemistry
mIHC: multiplex immunohistochemistry
H score: histochemistry score

## References

[1] Charo IF, Taub R (2011). Anti-inflammatory therapeutics for the treatment of atherosclerosis. Nat Rev Drug Discov.

[2] Libby P (2021). The changing landscape of atherosclerosis. Nature.

[3] Gimbrone MA Jr, García-Cardeña G (2016). Endothelial Cell Dysfunction and the Pathobiology of Atherosclerosis. Circ Res.

[4] Yu EP, Bennett MR (2014). Mitochondrial DNA damage and atherosclerosis. Trends Endocrinol Metab.

[5] Song P, Fang Z, Wang H, Cai Y, Rahimi K, Zhu Y (2020). Global and regional prevalence, burden, and risk factors for carotid atherosclerosis: a systematic review, meta-analysis, and modelling study. Lancet Glob Health.

[6] North BJ, Sinclair DA (2012). The intersection between aging and cardiovascular disease. Circ Res.

[7] Ali I, Zhang H, Zaidi SAA, Zhou G (2024). Understanding the intricacies of cellular senescence in atherosclerosis: Mechanisms and therapeutic implications. Ageing Res Rev.

[8] Katzir I, Adler M, Karin O, Mendelsohn-Cohen N, Mayo A, Alon U (2021). Senescent cells and the incidence of age-related diseases. Aging Cell.

[9] Childs BG, Baker DJ, Wijshake T, Conover CA, Campisi J, van Deursen JM (2016). Senescent intimal foam cells are deleterious at all stages of atherosclerosis. Science.

[10] Stojanović SD, Fiedler J, Bauersachs J, Thum T, Sedding DG (2020). Senescence-induced inflammation: an important player and key therapeutic target in atherosclerosis. Eur Heart J.

[11] Bennett MR, Clarke MC (2017). Killing the old: cell senescence in atherosclerosis. Nat Rev Cardiol.

[12] Zeng Q, Gong Y, Zhu N, Shi Y, Zhang C, Qin L (2024). Lipids and lipid metabolism in cellular senescence: Emerging targets for age-related diseases. Ageing Res Rev.

[13] Xiang Q, Tian F, Xu J, Du X, Zhang S, Liu L (2022). New insight into dyslipidemia-induced cellular senescence in atherosclerosis. Biol Rev Camb Philos Soc.

[14] Li X, Chen M, Chen X, He X, Li X, Wei H (2024). TRAP1 drives smooth muscle cell senescence and promotes atherosclerosis via HDAC3-primed histone H4 lysine 12 lactylation. Eur Heart J.

[15] Wang J, Uryga AK, Reinhold J, Figg N, Baker L, Finigan A (2015). Vascular Smooth Muscle Cell Senescence Promotes Atherosclerosis and Features of Plaque Vulnerability. Circulation.

[16] Grootaert MOJ, Finigan A, Figg NL, Uryga AK, Bennett MR (2021). SIRT6 Protects Smooth Muscle Cells from Senescence and Reduces Atherosclerosis. Circ Res.

[17] Fernandez DM, Giannarelli C (2022). Immune cell profiling in atherosclerosis: role in research and precision medicine. Nat Rev Cardiol.

[18] Yu Y, Li Y, Zhou L, Cheng X, Gong Z (2024). Hepatic stellate cells promote hepatocellular carcinoma development by regulating histone lactylation: Novel insights from single-cell RNA sequencing and spatial transcriptomics analyses. Cancer Lett.

[19] Tamargo IA, Baek KI, Xu C, Kang DW, Kim Y, Andueza A (2024). HEG1 Protects Against Atherosclerosis by Regulating Stable Flow-Induced KLF2/4 Expression in Endothelial Cells. Circulation.

[20] Dong Y, Wang B, Du M, Zhu B, Cui K, Li K (2023). Targeting Epsins to Inhibit Fibroblast Growth Factor Signaling While Potentiating Transforming Growth Factor-β Signaling Constrains Endothelial-to-Mesenchymal Transition in Atherosclerosis. Circulation.

[21] Pan H, Xue C, Auerbach BJ, Fan J, Bashore AC, Cui J (2020). Single-Cell Genomics Reveals a Novel Cell State During Smooth Muscle Cell Phenotypic Switching and Potential Therapeutic Targets for Atherosclerosis in Mouse and Human. Circulation.

[22] Traeuble K, Munz M, Pauli J, Sachs N, Vafadarnejad E, Carrillo-Roa T (2025). Integrated single-cell atlas of human atherosclerotic plaques. Nat Commun.

[23] Bleckwehl T, Babler A, Tebens M, Maryam S, Nyberg M, Bosteen M (2025). Encompassing view of spatial and single-cell RNA sequencing renews the role of the microvasculature in human atherosclerosis. Nat Cardiovasc Res.

[24] Xiao X, Xu M, Yu H, Wang L, Li X, Rak J (2021). Mesenchymal stem cell-derived small extracellular vesicles mitigate oxidative stress-induced senescence in endothelial cells via regulation of miR-146a/Src. Signal Transduct Target Ther.

[25] Hu G, Zhou H, Yuan Z, Wang J (2025). Vascular cellular senescence in human atherosclerosis: The critical modulating roles of CDKN2A and CDK4/6 signaling pathways. Biochem Pharmacol.

[26] Kang CM, Zhao JJ, Xie XX, Yu KW, Lai BC, Wang YX (2025). Unveiling the role of GATA4 in endothelial cell senescence and atherosclerosis development. Atherosclerosis.

[27] Ha GH, Park JS, Breuer EK (2013). TACC3 promotes epithelial-mesenchymal transition (EMT) through the activation of PI3K/Akt and ERK signaling pathways. Cancer Lett.

[28] Wang X, Li S, Liu C, Zhao J, Ren G, Zhang F (2024). High expression of PLA2G2A in fibroblasts plays a crucial role in the early progression of carotid atherosclerosis. J Transl Med.

[29] Kumar S, Nanduri R, Bhagyaraj E, Kalra R, Ahuja N, Chacko AP (2021). Vitamin D3-VDR-PTPN6 axis mediated autophagy contributes to the inhibition of macrophage foam cell formation. Autophagy.

[30] Kahr WH, Pluthero FG, Elkadri A, Warner N, Drobac M, Chen CH (2017). Loss of the Arp2/3 complex component ARPC1B causes platelet abnormalities and predisposes to inflammatory disease. Nat Commun.

[31] Chang X, Wang B, Zhao Y, Deng B, Liu P, Wang Y (2024). The role of IFI16 in regulating PANoptosis and implication in heart diseases. Cell Death Discov.

[32] Chu VT, Enghard P, Riemekasten G, Berek C (2007). In vitro and in vivo activation induces BAFF and APRIL expression in B cells. J Immunol.

[33] Sandberg WJ, Otterdal K, Gullestad L, Halvorsen B, Ragnarsson A, Frøland SS (2009). The tumour necrosis factor superfamily ligand APRIL (TNFSF13) is released upon platelet activation and expressed in atherosclerosis. Thromb Haemost.

[34] Yamada Y, Sakuma J, Takeuchi I, Yasukochi Y, Kato K, Oguri M (2017). Identification of TNFSF13, SPATC1L, SLC22A25 and SALL4 as novel susceptibility loci for atrial fibrillation by an exome-wide association study. Mol Med Rep.

[35] Al-Mubarak AA, Markousis Mavrogenis G, Guo X, De Bruyn M, Nath M, Romaine SPR (2024). Biomarker and transcriptomics profiles of serum selenium concentrations in patients with heart failure are associated with immunoregulatory processes. Redox Biol.

[36] Tsiantoulas D, Eslami M, Obermayer G, Clement M, Smeets D, Mayer FJ (2021). APRIL limits atherosclerosis by binding to heparan sulfate proteoglycans. Nature.

[37] Salminen A, Kauppinen A, Kaarniranta K (2012). Emerging role of NF-κB signaling in the induction of senescence-associated secretory phenotype (SASP). Cell Signal.

[38] Sun Y, Wang X, Liu T, Zhu X, Pan X (2022). The multifaceted role of the SASP in atherosclerosis: from mechanisms to therapeutic opportunities. Cell Biosci.

[39] Lee SM, Kim EJ, Suk K, Lee WH (2011). BAFF and APRIL induce inflammatory activation of THP-1 cells through interaction with their conventional receptors and activation of MAPK and NF-κB. Inflamm Res.

[40] Fernandez DM, Rahman AH, Fernandez NF, Chudnovskiy A, Amir ED, Amadori L (2019). Single-cell immune landscape of human atherosclerotic plaques. Nat Med.

[41] Gao J, Shi L, Gu J, Zhang D, Wang W, Zhu X (2021). Difference of immune cell infiltration between stable and unstable carotid artery atherosclerosis. J Cell Mol Med.

[42] Cheng HY, Wu R, Hedrick CC (2014). Gammadelta (γδ) T lymphocytes do not impact the development of early atherosclerosis. Atherosclerosis.

[43] Stark MA, Huo Y, Burcin TL, Morris MA, Olson TS, Ley K (2005). Phagocytosis of apoptotic neutrophils regulates granulopoiesis via IL-23 and IL-17. Immunity.

[44] Ouimet M, Ediriweera H, Afonso MS, Ramkhelawon B, Singaravelu R, Liao X (2017). microRNA-33 Regulates Macrophage Autophagy in Atherosclerosis. Arterioscler Thromb Vasc Biol.

[45] Boucher DM, Robichaud S, Lorant V, Leon JS, Suliman I, Rasheed A (2025). Age-Related Impairments in Immune Cell Efferocytosis and Autophagy Hinder Atherosclerosis Regression. Arterioscler Thromb Vasc Biol.

