# Supplementary material for: TNFSF13 Drives Atherosclerosis Progression by Targeting Macrophage Senescence
Source: Int J Med Sci. 2026 Jul 22;23(9):2780–95. doi: 10.7150/ijms.130617 (PMC13539914; doi:10.7150/ijms.130617)
Supplement: Supplementary file 1 [file ijmsv23p2780s1.pdf]

## *Supplementary table*

Table 1. The details of three databases.

| Dataset | Platform | Samples | |
|---|---|---|---|
| GSE28829 | GPL570 | 13 early AS plaque samples | 16 advanced AS plaque samples |
| GSE100927 | GPL17077 | 35 non-atherosclerotic patients | 69 atherosclerotic patients |
| GSE163154 | GPL6104 | 16 non-IPH samples | 27 IPH samples |

*IPH: intraplaque hemorrhage. non-IPH: Non-intraplaque hemorrhage

Table 2. The forward-primer and the reverse-primer of TNFSF13 and GAPDH.

| Name | Forward-primer | Reverse-primer |
|---|---|---|
| TNFSF13 | 5 '-CAGAGCCTCAGGAGAGAGGT-3 ' | 5 '-GCAGGACAGAGTGCTGCT-3 ' |
| GAPDH | 5 '-CTGGGCTACACTGAGCACC-3 ' | 5 '-AAGTGGTCGTTGAGGGCAATG-3 ' |

Table 3.The detail of tissue collection timeline.

| Sample ID | Tissue type | Clinical Diagnosis | Collection Date |
|---|---|---|---|
| Nor-01 | left coronary artery | Normal | 2022-07-23 |
| Nor-02 | left coronary artery | Normal | 2022-04-04 |
| Nor-03 | left coronary artery | Normal | 2022-03-04 |
| Nor-04 | left coronary artery | Normal | 2022-01-24 |
| AS-01 | left coronary artery | Atherosclerosis | 2024-04-04 |
| AS-02 | left coronary artery | Atherosclerosis | 2024-04-10 |
| AS-03 | left coronary artery | Atherosclerosis | 2024-04-18 |
| AS-04 | left coronary artery | Atherosclerosis | 2024-04-21 |

## *Additional Figure*

A

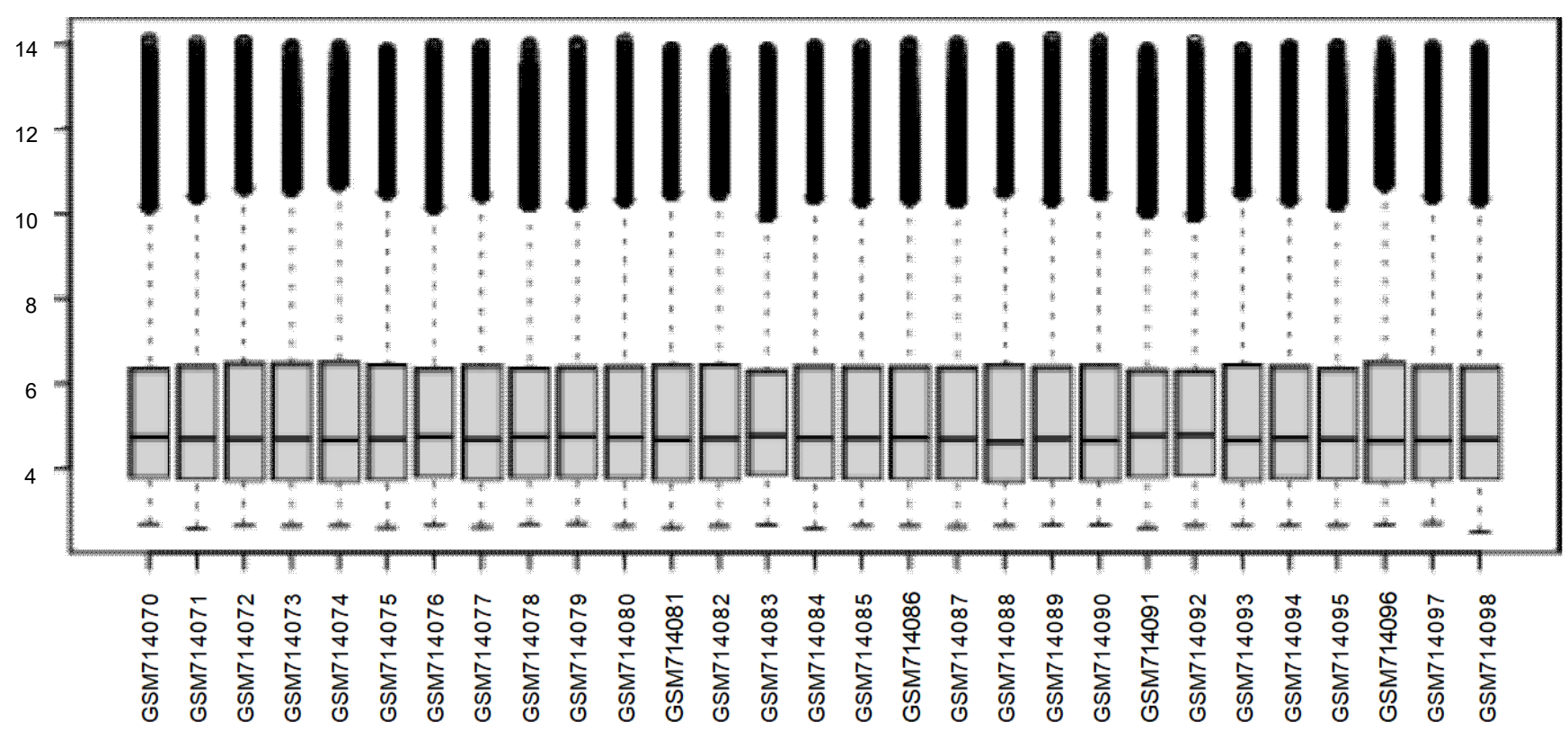

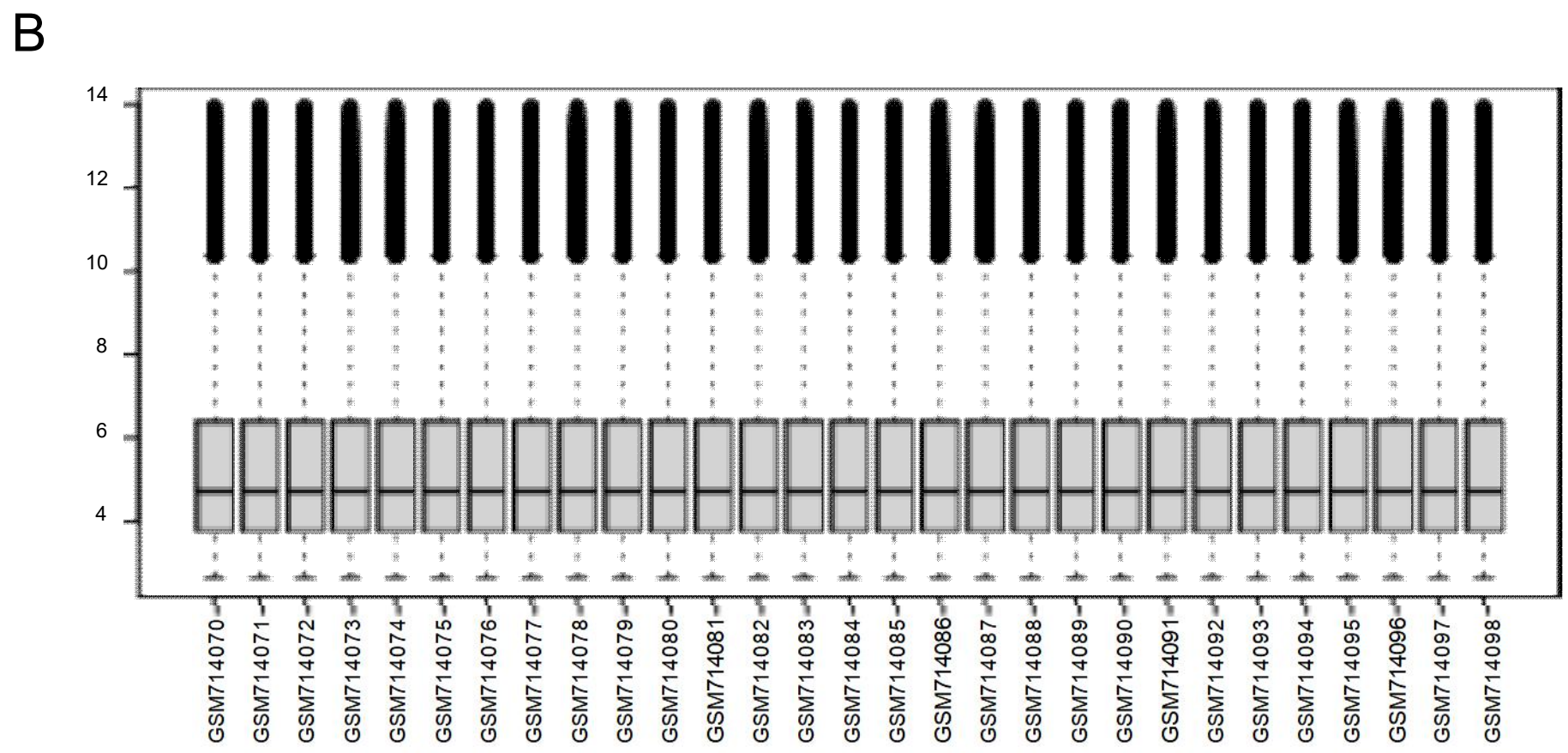


**Figure S1. B oxplot of GSE28829 before and after normalization (A)** boxplot of GSE28829 before normalization. **(B)** boxplot of GSE28829 after normalization.

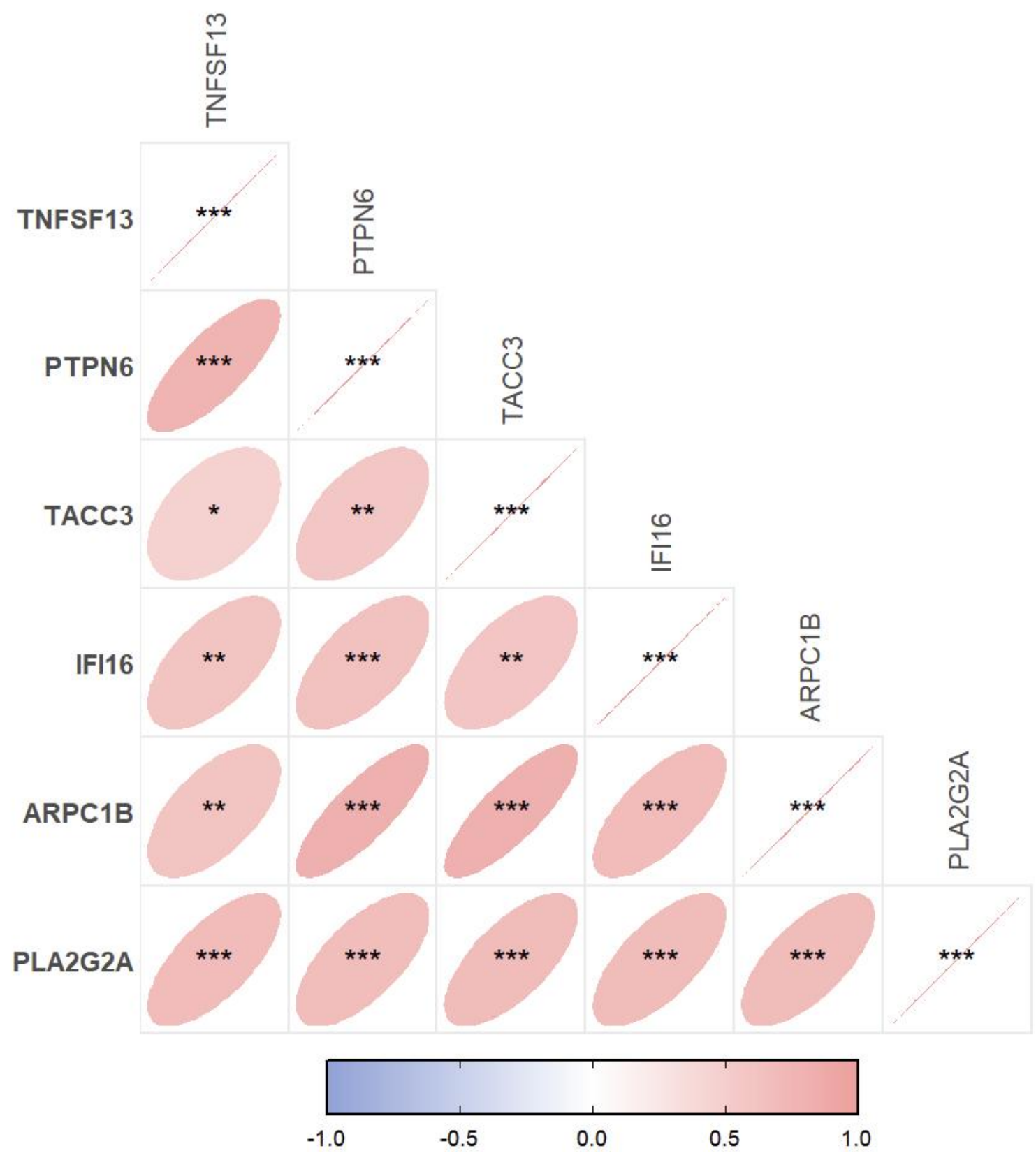


**Figure S2. The correlation heatmap between six hub genes.** Heatmap showing the correlation analysis between the 6 hub genes. The size of the ellipses represent the strength of the correlation coefficients. Red and blue indicate positive and negative correlations, respectively.
